# Not Just Pictures: Utility of Camera Trapping in the Context of African Swine Fever and Wild Boar Management

**DOI:** 10.1155/2023/7820538

**Published:** 2023-02-25

**Authors:** Pablo Palencia, Rachele Vada, Stefania Zanet, Mara Calvini, Andrea De Giovanni, Giacomo Gola, Ezio Ferroglio

**Affiliations:** ^1^Università Degli Studi di Torino, Dipartamiento di Scienze Veterinarie, Largo Paolo Braccini 2, Grugliasco, Torino 10095, Italy; ^2^Ente di Gestione Delle Aree Protette Dell'Appennino Piemontese, Via Umberto I 51, Salita Poggio, Bosio 15060, Italy

## Abstract

African swine fever (ASF) is a highly contagious disease affecting all suids and wild boar (*Sus scrofa*). Since 2007, ASF has spread to more than 30 countries in Europe and Asian regions, and the most recent outbreak has been in mainland Italy (reported on January 2022). When the genotype II of the ASF virus infects a population, a mortality rate close to 90% is usually reported. This drop in wild boar abundance produces a cascade effect in the entire ecosystem. In this context, effective monitoring tools for deriving management parameters are a priority aspect, and the utility of camera trapping could have been overlooked. Here, sampling the infected area in north Italy, we showed the utility of camera traps in the context of ASF infection. Specifically, we used 43 camera traps randomly distributed to (i) estimate movement parameters and population density of wild boar, roe deer (*Capreolus capreolus*), and wolf (*Canis lupus*); (ii) quantify wild boar recruitment; and (iii) assess whether the human restriction rules are being met. On the first spring after the outbreak detection, our results for wild boar indicated a density of 0.27 ind·km^−2^ ± 0.11 (standard error, SE), a daily activity level of 0.49 ± 0.07 (i.e., 11.76 h·day^−1^), a daily distance travelled of 9.07 ± 1.80 km·day^−1^, a litter size of 1.72 piglets·group^−1^, and a 72% of pregnant females. Despite human outdoor activities being restricted in the infected zone, we recorded human presence in 19 camera traps. The wide range of parameters estimated from the camera trap data, together with some intrinsic and practical advantages of this tool, allows us to conclude that camera traps are well positioned to be a reference approach to monitor populations affected by ASF. The population-specific parameters are of prime importance for optimizing ASF control efforts.

## 1. Introduction

The African swine fever (ASF) epidemic is caused by a lethal viral hemorrhagic disease of swine (ASFV) that infects both wild and domestic suids and causes a mortality rate of about 90% within 4 to 15 days postinfection [1]. ASF is affecting Eurasia, and it is transmitted by direct contact between infected animals and by ingestion of infected pork or other contaminated materials, been blood contact (carcass-based) the most efficient means of transmission [2, 3]. In Eurasia, suitable soft ticks are either absent or not relevant for ASF transmission, but the role of other mechanical transmissions such as clothing, transport trucks, or feed supplies has been discussed [2]. Since 2007, when the virus was first observed in Georgia, the ASFV has spread to more than 30 countries in the European and Asian regions [4]. In brief, the virus spread quickly to neighbouring countries, and ASFV genotype II has been notified in Belarus, Belgium, Bulgaria, Czechia, Estonia, Germany, Greece, Hungary, Italy, Latvia, Lithuania, Moldova, North Macedonia, Poland, Romania, Russia, Serbia, Slovakia, and Ukraine [4]. Belgium became free from ASF in 2020, Czechia in 2019, and the Baltic states showed a declining trend in polymerase chain reaction-positive wild boar carcasses [4, 5]. The ongoing ASF pandemic is unprecedented in its geographical spread and impact on the global pork industry, and nowadays its management is a priority aspect at a global level [4] and wild suid populations [6]. In many ASF-affected countries of Eurasia, wild boar (*Sus scrofa*) is a relevant wildlife host for the ASF virus, contributing to infection, maintenance, and spread, representing a notable challenge for disease control [5].

Wild boar is a widespread native Palearctic ungulate whose population has sharply increased in the last decades [7]. Because of both natural expansion and human introductions, the wild boar now occurs in all continents except Antarctica [8]. This species is involved in diverse and increasing conflicts, such as those related to damage to agriculture, collisions with vehicles, environmental effects, and the transmission of shared infections [9]. Moreover, the wild boar populations have also expanded spatially, overlapping with areas of livestock production [10]. In outdoor pig farms, wild boar-domestic pig interactions usually occur and thus the risk of maintenance and spread of ASF [11].

In this context, monitoring wild boar populations for effective ASF management is a priority aspect. Wild boar is an elusive species with nocturnal activity patterns. These behavioural characteristics limited the utility of most traditional wildlife monitoring methods that are usually based on the direct observation of the animals [12]. However, during the last decades, the use of remotely activated cameras (i.e., camera traps) has overcome most of these limitations and has been frequently described as a reliable and cost-efficient monitoring method [13]. Regarding the utility of camera traps in the context of ASF pandemic, three studies have been already published. Cadenas-Fernández et al. [14] examined the interaction between free-ranging pigs and wild boar in an ASF-endemic area of Sardinia; Morelle et al. [6] evaluated the disease-induced mortality due to ASF in Poland; and Bollen et al. [15] assessed the spatiotemporal variability in wild boar occupancy in infected and noninfected zones in Belgium. To the best of our knowledge, these are the only three studies that have used camera traps in the context of wild boar and the ASF pandemic.

Other relevant applications of camera trapping in the ASF context are still underexplored. First, camera trapping is a noninvasive methodology, and thus a minimum disturbance is caused in the sampled population, which is a priority aspect when an outbreak is reported in a population [16]. Camera traps can provide useful data regarding the spread, persistence, and impact of the virus. Regarding the spread, it is well described that the vast majority of ASF outbreaks are mediated by human movements, for instance, by pork-contaminated products and/or transporting the virus by clothes [17, 18]. Thus, human restriction laws and prohibiting outdoor activities are habitually implemented. Camera traps can provide evidence of the violation of the laws in areas difficult to control by other methods. Camera traps can be also applied to estimate movement parameters such as activity patterns and daily distances travelled [19–21]. Regarding disease persistence, it has been described that spring recruitment (expressed as the proportion of females giving birth to piglets) has a strong effect on ASF persistence [22]. Newly born wild boar is susceptible to infection and virus transmission [22]. Camera traps can provide reliable data on piglet presence and litter size [23]. Finally, regarding ASF impacts, recently described multispecies methodologies could be useful to derive population parameters such as abundance, not only of wild boar but also of other mammals indirectly affected (and involved) by ASF outbreaks [24].

In this context, we aimed to provide further insights into the utility of camera traps to monitor wild boar populations affected by an ASF outbreak. Especially, we targeted (i) to estimate movement parameters such as the proportion of the day that the population spend in movement and the average daily distances movement by the individuals; (ii) to provide robust and reliable population density estimates of not only wild boar but also other mammal's species with a relevant role under a scenario of ASF outbreak (e.g., roe deer (*Capreolus capreolus*) and wolf (*Canis lupus*)); (iii) to quantify spring recruitment; and (iv) to provide empirical evidence of compliance with the rules of human movement restriction. For that, we considered a case study of the recently infected area in north Italy [18], where not essential outdoor activities were restricted [25].

## 2. Materials and Methods

### 2.1. Study Area

**Figure 1:**
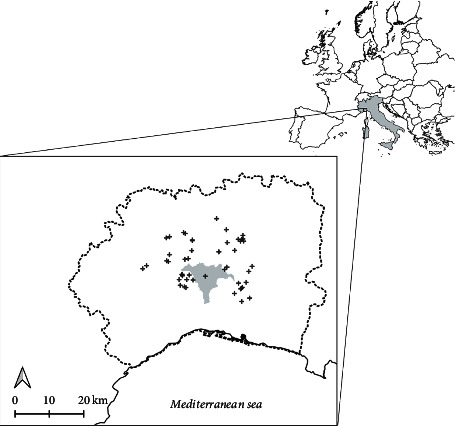
Location of the study area in northern Italy. Crosses represent positive African swine fever (ASF) wild boar reported; the dashed line represents the zone II of restriction according to administrative zonation due to ASF emergence; and the grey polygon in the bottom panel represents the area surveyed in this study placed in the core of the infected area.

The study was carried out in the Natural Park Capanne di Marcarolo National Park, a territory of 8288 ha located in north Italy (Alessandria province, the Piedmont region) (Figure 1). It is a mountainous area, with altitudes ranging from 335 to 1170 m.a.s.l (average 700 m.a.s.l.). The vegetation is characterized by sparse wood of oaks (*Quercus petraea*), beeches (*Fagus sylvatica*) and pines (*Pinus pinaster* and *Pinus silvestris*), and dry grasslands. A total checklist of 955 plant species with 3 strict endemic ones (*Aquilegia ophiolithica*, *Cerastium utriense,* and *Viola bertolonii*) has been described [26]. The climate is continental-temperate with marked seasons and annual precipitation ca. 1500 mm. The management team of the natural park authorized and supported the study.

### 2.2. Field Methods

From March to August 2022, 43 camera traps (Browning Strike Force HD X Pro-model BTC-5HDPX) were randomly deployed covering the study area (Figure 1). Cameras were deployed facing north, 50 cm above the ground, with the sensor angled parallel to the slope, and fixed to the tree with a steel cable for security reasons. Realised sampling locations deviated from the design by as much as 100 m to mount camera traps on trees and avoided very unfavourable conditions (e.g., high slopes in front of the camera). Only one camera was pointed out at a human trail. Cameras were set to be operative all day, to record a burst of eight consecutive pictures (rapid fire settings) at each activation, with the minimum time lapse (0.22 sec) between consecutive activations. Nocturnal pictures were illuminated with infrared flash (low glow). A table describing camera trap settings can be found in Appendix S1. Neither baits nor attractants were used. The date and time of each capture were automatically stamped onto each picture. Once a month, we checked the battery and memory status of the cameras. After each fieldwork session, boots, clothes, and materials were disinfected with a Virkon S solution.

To lastly estimate the location of the photo-captured animals in the field of view of the camera (see details below), we used a photogrammetry method [27]. Thus, on each deployment (43 field stations), we recorded 20 pictures of a 1 m length pole with marks at 20 cm intervals. These 20 pole locations were randomly distributed within the camera field of view, covering the entire viewing angle (55° in our devices) and upto a maximum distance of 15 m from the camera. The pole was held perpendicular to the camera's passive infrared sensor. These 20 photos were taken before and after each camera was checked to evaluate battery and memory status. Additionally, to apply this photogrammetry method, it is necessary to calibrate the camera trap model. The camera model was calibrated once in a flat area without dense vegetation. We used the same 1 m pole used on the field to calibrate the deployments. In this case, the pole was set at known distances. Specially, we defined four distances (2.5, 5, 7.5, and 10 m) from the camera, and we placed 6 times on each distance the pole, covering the viewing angle. Thus, we recorded 24 pictures of the pole at known distances to the camera.

### 2.3. Analysis

#### 2.3.1. Movement Patterns

Wild boar but also wolves' movements have been described as the potential drivers of ASF spread by faces and/or mechanical transportation of the virus with mud on the paws [28, 29]. Camera trap-based methodologies have been described to estimate (i) activity level (i.e., the proportion of the day that the population spend in movement) and daily distance travelled (i.e., day range) of wildlife. From camera trap data, the activity level is estimated by fitting circular kernel distribution to the time in which the animals were recorded [19]. The day range is estimated by multiplying the activity level by the average speed of movement [20] and accounting for different movement behaviours [21]. Movement speed was estimated by dividing the distance travelled by each individual photo-captured by the time elapsed. Individuals reacting to the camera were discarded from the analysis as their natural behaviour was modified by our sampling method. Animal positions to derive speed density were estimated by the photogrammetry methods and using CTtracking tools [27]. In brief, we tracked the pixel position of each individual in each picture. Then, using the calibration imagery, we converted each pixel position into radial and angular distances from the camera. These distances were trigonometrically transformed to estimate the distance moved across each encounter and then estimate the speed of movement by accounting for the time elapsed (see [27] for a more in detail explanation). The presence of different movement behaviours in the sampled population was explored by fitting log-normal mixture models using the R package trappingmotion and following the procedure described by [21].

#### 2.3.2. Mammal's Population Density

The random encounter model (REM) [30] was applied to estimate the population density of the target species (i.e., wild boar, roe deer, and wolf). The REM has been validated as a reliable method to estimate population density when individual identification is not possible [24] and has been applied as a reference method to monitor wild boar density [31–34]. It has also been evidenced the utility of the REM to estimate the population density of the community of mammals inhabiting an area [24, 27]. The REM models the random encounters between animals and cameras and estimates density accounting for other factors that affect the encounter rate (i.e., animal movement and camera trap detection capability). The REM equation is as follows:

(1) $$\begin{array}{c} D=\frac{y}{t}\cdot \frac{\pi }{v\cdot r\cdot \left(2+Ɵ\right)}\text{,} \end{array}$$

where *y* is the number of encounters, *t* is the total survey effort, *v* is the day range, and *r* and *Ɵ* refer to the effective radius and angle of the camera detection zone, respectively. An individual of the target species entering the detection zone was considered an encounter (*y*). As our sampling period overlapped with the calving season, we discarded year-born individuals from the analysis, and thus we estimated the density of adult (>1 year) individuals. To estimate *t*, we quantified the total number of days that each camera was operative during the sampling periods. We discarded those camera days in which a given camera was not operative due to empty batteries, full memory, and/or stolen. To estimate the detection zone, we only considered the animal's position in the first picture in which each one was recorded. Distances and angles for the first detection were modelled under distance sampling theory to finally estimate the effective detection zone size [35, 36].

The R packages activity, distance and trappingmotion, and some functions in CTtracking repository (https://github.com/MarcusRowcliffe/CTtracking) were used. The R code, working data, and vignette to run a REM analysis are available in the following link https://github.com/PabloPalencia/CameraTrappingAnalysis/tree/main/REM.

#### 2.3.3. Spring Recruitment

We selected an interval longer than 10 min to consider two consecutive records of wild boar as independent events. After the first record of a group of piglets, we recorded in each event (i) the sex of the adult individuals on the basics of external signs and (ii) the litter size (number of piglets) when present.

#### 2.3.4. Biosafety: Human Movement Restriction

Using the abovementioned 43 camera traps deployment, we evaluated the presence of humans and domestic dogs in the pictures recorded during the entire sampling period. Those deployments in which the camera was stolen were quantified as positive to human presence despite we did not have the photographic evidence. On every single camera, we also quantified the time gap between a human and the previous wild boar encounter.

## 3. Results

A total of 980,883 pictures were recorded during an effective sampling effort of 4037 camera·days. A total of 12 mammal species were recorded. Roe deer was the more frequent, with more than 1500 encounters, followed by the European hare (*Lepus europaeus*) with 228 encounters. We also detected other rare and endangered species such as wild cat (*Felis silvestris*), polecat (*Mustela putorius*), and weasel (*Mustela nivalis*). See a summary of all the mammals recorded in Appendix S2 and the row data to replicate the analysis in Appendix S3.

### 3.1. Movement Patterns

**Figure 2:**
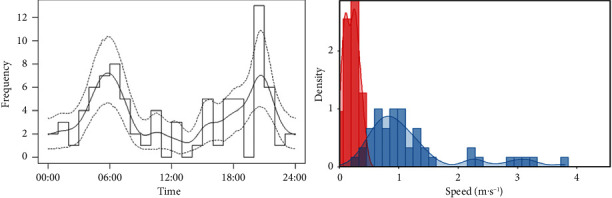
Movement parameters for wild boar. The left panel represents the activity pattern (continuous line being the mean, dashed line being 95% confidence interval). The right panel represents the two movement patterns observed in the population. The red colour represents slow movements (e.g., feeding), and the blue colour represents fast movements (e.g., displacement between habitat patches).

The activity level was 0.54 ± 0.06 (standard error, SE) for roe deer, 0.49 ± 0.07 for wild boar, and 0.60 ± 0.10 for wolf (Figure 2). This indicates that the roe deer spent 12.96 hours active per day, wild boar spent 11.76 hours, and wolf spent 14.4 hours. Regarding the day range, the roe deer showed the lowest daily displacement (4.64 km·day^−1^ ± 0.97), 9.07 km·day^−1^ ± 1.80 for wild boar, and 37.74 km·day^−1^ ± 6.91 for wolves. While two movement behaviours were observed in wild boar (mean speed slow behaviour: 0.09 m·s^−1^ ± 0.03; fast behaviour: 0.87 ± 0.08), only one behaviour was observed in roe deer (0.10 m·s^−1^ ± 0.02) and wolf (0.73 m·s^−1^ ± 0.10) (Figure 2).

### 3.2. Mammals' Population Density

Estimated densities were 0.27 ind·km^−2^ ± 0.11 for wild boar, 6.35 ind·km^−2^ ± 1.88 for roe deer, and 0.07 ind·km^−2^ ± 0.03 for wolf.

### 3.3. Spring Recruitment

The first evidence of wild boar reproduction was recorded on May, 6^th^, when we recorded a female with three piglets in one camera. After that, we recorded 72 independent wild boar events of one or two adult individuals (average 1.26 ind·group^−1^). Specifically, we recorded 17 events of males, 41 events of females and piglets (24 of them only piglets), and 14 events in which we were not able to identify sex. From the 41 events of females and piglets, we recorded piglets in 32 of them, with an average little size of 1.72 piglets·group^−1^ (min: 1; max: 4).

### 3.4. Human Restriction Laws

**Figure 3:**
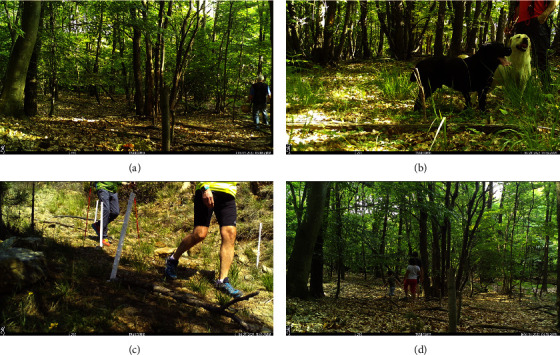
Example of pictures evidencing human presence and domestic dogs off leash in an area restricted to human movements due to the African swine fever outbreak. Different outdoor activities were recorded, such as mushroom picking (a), walking the dog (b), trekking (c), and family walks (d).

Human presence was detected in 19 camera traps, and seven of them were stolen. A maximum of 14 people was detected in a single camera. Domestic dogs, always off leash, were detected in eight cameras. Humans were detected doing different open-air leisure activities such as trekking, mushroom picking, family walks, or walking the dog (Figure 3). Wild boar presence was confirmed in 50% of the cameras in which humans were observed. Except for one wild boar-human interaction spaced by 125 days, all the others occurred in less than 12 days (mean = 6.4), with a minimum time gap of 12 h.

## 4. Discussion

In this study, we evidenced the utility of camera traps to monitor populations infected with ASFV by reporting both ecological and management-related parameters.

The ASFV (genotype II) was first detected in mainland Italy on 29 December, 2021, in a male adult wild boar. In the next few days, other wild boars were found dead in a buffer zone of 20 km [18]. In 2022, other two outbreaks have been reported in central Italy (farther than 500 km from the first report) and the spill-over to domestic pig farms occurred. These long-distance jumps reinforced the hypothesis that the transmission occurred via the human factor. Emergency measures to prevent the spread of the diseases were undertaken by Italian authorities, especially focused on passive surveillance and biosecurity measures, fencing, and human activity regulation [25]. Focusing on human restrictions, hunting and other outdoor activities were prohibited in the infected area and neighbouring regions (Figure 1). Concerning this, our camera traps provided evidence of a restriction violation. Human pictures were present in 19 camera deployments, showing a great variety of outdoor activities such as picking up mushrooms, trekking, and/or family walks (Figure 3). The human presence was frequent in some cameras, with a maximum of 14 people recorded in one camera. Additionally, free-ranging dogs were also observed (Figure 3). Previous studies have shown that the survival times of the virus on the soil strongly depend on the type of soil (e.g., sand vs. humus-rich one), with average survival times ranging from less than three days to upto two weeks [37]. In contaminated water, the infectious virus was detectable for more than two weeks [37]. Here, the average time gap between a wild boar and the subsequent human encounter was 6.4 days, with some encounters spaced just 12 h. Thus, it has been possible the mechanical spread of the virus by clothing or mud rest [17]. We would like to emphasize that all the cameras except one were not pointing to human trails; thus, we expect that the human presence in the infected (and restricted) area should be more frequent in hiking trails and paths. Additionally, humans usually avoid being recorded by the cameras by passing behind them. Thus, the human presence in the study area could be even underestimated. Despite other actions such as 270 km of fencing has been implemented to control the spread of the virus, allocating funds to implement vigilance for restriction rules and increasing dissemination campaigns to stakeholders and the general public are instead needed to prevent the spread of the virus according to our results. It has been demonstrated that fencing is neither the most effective action for widely affected areas [4, 38, 39] nor to control the movements of wild boar [40].

Regarding the estimation of ecological parameters, camera traps have been broadly used to monitor different ecological, behavioural, and sanitary aspects of wild boar. Specifically, it has been used to monitor population densities and abundance [33, 41, 42]; movement parameters such as activity level and daily distance travelled [21, 43, 44]; species interactions [14, 45, 46]; reproduction success [23]; and diseases transmission risk points [11, 47] among others. Even more recently, some studies have discussed the utility of camera trapping to be implemented in integrated wildlife monitoring programs targeted at wildlife health and host community monitoring [48]. Here, we reinforced the utility of camera traps in the ASF epidemic [6, 14, 15]. Despite the management of ASF is usually focused on the wild boar and pork industry [49], the ecological impact of ASF emergence in a wild boar population could affect the entire ecosystem. Wild boar is considered an “ecosystem engineering” [50], and a drastic decrease in wild boar abundance due to ASF high mortality is expected to cause significant changes. For instance, a recent study described that wolves' diet changed to more predation on both roe and red deer after ASF emergence in Belarus [51]. Similarly, the emergence of ASF in Poland derived in a higher number of livestock attacks by wolves [52]. Thus, the utility of multispecies methodologies to monitor both wild boar population size but also other species involved in the dynamic of the ecosystem will improve the better understanding of the system as well as the management actions implemented [53]. The random encounter model is a camera trap-based method that allows the estimation of population density without the need to individually identify animals [30] and spatial autocorrelation in the camera trap placements. This provides the flexibility of a given survey design to estimate the density of more than one species [24, 27, 30, 32, 33]. Reliable data on the abundance of multiple species after an ASF outbreak will improve decision-making. To the best of our knowledge, this is the first study in which the wild boar, other ungulates (here roe deer), and main predator (here wolf) population densities have been estimated. While the REM has been already applied and validated for wild boar and roe deer [24, 33, 34, 54], this is the first study in which the REM has been applied to estimate wolf density. The REM has not been yet tested against wolf reference densities; but, it has been previously applied to other carnivores and/or apex predators (*Felis silvestris*—[55]; *Panthera leo*—[56]; *Puma concolor*—[57]). The point density estimated here is equivalent to that reported by genetic sampling in the study area [58]. Regarding the wild boar density, a previous study based on the capture-recapture method estimated a density in the study area ranging from 3.45 to 6.90 ind·km^−2^ in 2006 [59]. According to the general population trend of wild boar in Europe [7], it is expected that the wild boar abundance has increased in the subsequent years till the ASF outbreak. Inside the study area, the first ASF-positive carcasses were reported in early January 2022 (Figure 1). Our sampling period covers from two to six months later; thus, the wild boar density reported here is expected to be one of the lower after ASF emergence and significantly lower than before the ASF emergence. Previous studies have reported a drop in wild boar density by 84% and 95% within one year following the ASF outbreak [6]. Subsequent monitoring programs could be recommended to monitor both the wild boar and other species populations.

Regarding the movement patterns, the activity level and the day range estimated for the target species are in the range of those reported in other areas [21, 24, 33, 34, 48, 54]. Wild boar movement has been suggested to be only responsible for local transmission [60, 61] and other pathways (such as the human ones discussed above) be more dominant in medium- and long-distance spread of the disease. Some authors have also discussed the effect of animal movement, especially wolves, which usually display longer movements [62]. Broadly, a better understanding of wildlife movement patterns will help to improve the prediction capability of the spread of the virus [63]. Daily distances travelled by wild boar are relatively higher than those estimated in other wild boar populations across Europe [34]. This is consistent with the longer movement observed in marked animals [59]. In the capture-recapture study carried out in 2006, four individuals have been recaptured far away, 15 km from the study area, with a maximum of one male found at 28 km and a female found at 25 km [59]. These movement patterns (together with a likely late detection and human presence in the infected area) could explain the wide affected area, currently more than 3000 km^2^ (Figure 1).

Finally, regarding spring recruitment, the values reported in our study (i.e., 1.72 average litter size and 78% of females giving birth to piglets) are in the range of those previously published [34]. Through modelling, it has been demonstrated that ASF persistence is significantly and positively influenced by spring recruitment [22]. While a recent review concluded that the average percentage of pregnant females is lower than 50% for all the age classes [34], the value observed in our population was much higher. We acknowledge that pseudoreplication issues (i.e., the same female and piglets group recorded more than once) could emerge when using camera traps and monitoring species without natural marks that allow individual identification. However, while these multiple records could inflate the sample size, bias is not expected in average litter size, not on a percentage of females with piglets. An increase in reproductive performance will also generate a higher population growth during the endemic phase, increasing the chances for a second epidemic wave [22].

Despite that it has not been addressed in this study, camera traps are also a cost-efficient tool to monitor wild boar and domestic pig interactions [45, 46] and wild boar occupancy [15]. Previous studies have already monitored wild boar-domestic pig interactions in the context of ASF and free-ranging pigs, concluding its utility and the need to include this interface in the epidemiological assessments of ASF [14]. Occupancy estimates derived from camera trap data will also be useful to better understand wild boar-environment relationships and to select those areas preferentially occupied when intervention actions such as strengthening passive surveillance or culling are implemented [64].

In conclusion, our study reinforced the utility of camera traps to monitor populations infected by ASFV. Despite the initial inversion (ca. 150–200 € per device), camera trapping is a cost-efficient methodology, especially from the long-term point of view. By now, only Czech Republic and Belgium have recovered the ASF-freedom status, being the endemic status the most frequent in Europe after the virus infected an area [4]. The noninvasiveness characteristics together with the multispecies potential are also relevant advantages in the ASF context. New analytical procedures in which individual recognition is not needed are also especially relevant for wild boar [20, 21, 30]. Broadly, camera traps provide reliable data that could be subsequently considered in prevention, control, and eradication plans. We encourage wildlife managers, administrators, and decision-makers to consider a camera trap as a reference tool to estimate population-specific parameters, which are of prime importance for optimizing ASF management efforts.

## Data Availability

The data used to support the findings of this study are included within the supplementary information file (Appendix S3).

## Supplementary Materials

Supplementary Materials Appendix S1: camera trap settings. Appendix S2: summary of species recorded. Appendix S3: raw camera trap data.
