# Supplementary material for: Not Just Pictures: Utility of Camera Trapping in the Context of African Swine Fever and Wild Boar Management
Source: Transbound Emerg Dis. 2023 Feb 25;2023:7820538. doi: 10.1155/2023/7820538 (PMC12017085; doi:10.1155/2023/7820538)
Supplement: Supplementary Materials — Appendix S1: camera trap settings. Appendix S2: summary of species recorded. Appendix S3: raw camera trap data. [file 7820538.f1.docx]

**Not just pictures: utility of camera trapping in the context of African swine fever and wild boar management**

Pablo Palencia^1^, Rachele Vada^1^, Stefania Zanet^1^, Mara Calvini^2^, Andrea De Giovanni^2^, Giacomo Gola^2^ & Ezio Ferroglio^1^

1. Università Degli Studi di Torino, Dipartamiento di Scienze Veterinarie, Largo Paolo Braccini, 2, 10095 Grugliasco Torino, Italy
2. Ente di gestione delle Aree Protette dell’Appennino Piemontese, Via Umberto I 51 – Salita Poggio, 15060 Bosio, Italy

*Corresponding author: Università Degli Studi di Torino, Dipartamiento di Scienze Veterinarie, Largo Paolo Braccini, 2, 10095 Grugliasco Torino, Italy. E-mail. [palencia.pablo.m@gmail.com](mailto:palencia.pablo.m@gmail.com)

Appendix S1: Camera trap settings.

**Table 1 S1**. Camera traps settings used to increase the utility of camera traps in the context of African swine fever emergence. Settings are specific for Browning Strike Force HD X Pro – model BTC-5HDPX, but note that similar options can be in other brands and/or models.

| **Setting** | **Chosen** | **Reason** |
| --- | --- | --- |
| Capture timer | Off | We were focused on different species with different activity patterns. Thus, the cameras should be active 24 hours per day |
| Capture mode | Pictures | Rapidfire bursts of pictures are useful to reconstruct the trajectory of the animals when crossing the field of view. Pictures have some practical advantages in terms of cost-effectiveness in relation to videos (e.g. optimized memory usage, less time-consuming to process). |
| Capture delay | 1 sec | As lower as possible. Increase the probability of multiple activations in the same encounter |
| Picture size | 4 MP | As lower as possible. Optimize memory usage and is high enough to differentiate species |
| Multishot mode | 8 Rapid fire | Allows rapid image capture, and then increases the accuracy of animal position in the field of view |
| Adjustable IR flash | Power save | It is well described that the effective detection distance for wild boar and mammals is usually lower than 10 meters. “Power save” intensity illuminated properly medium-size mammals at this range of distances. |
|  |  |  |

Appendix S2: Summary of species recorded

**
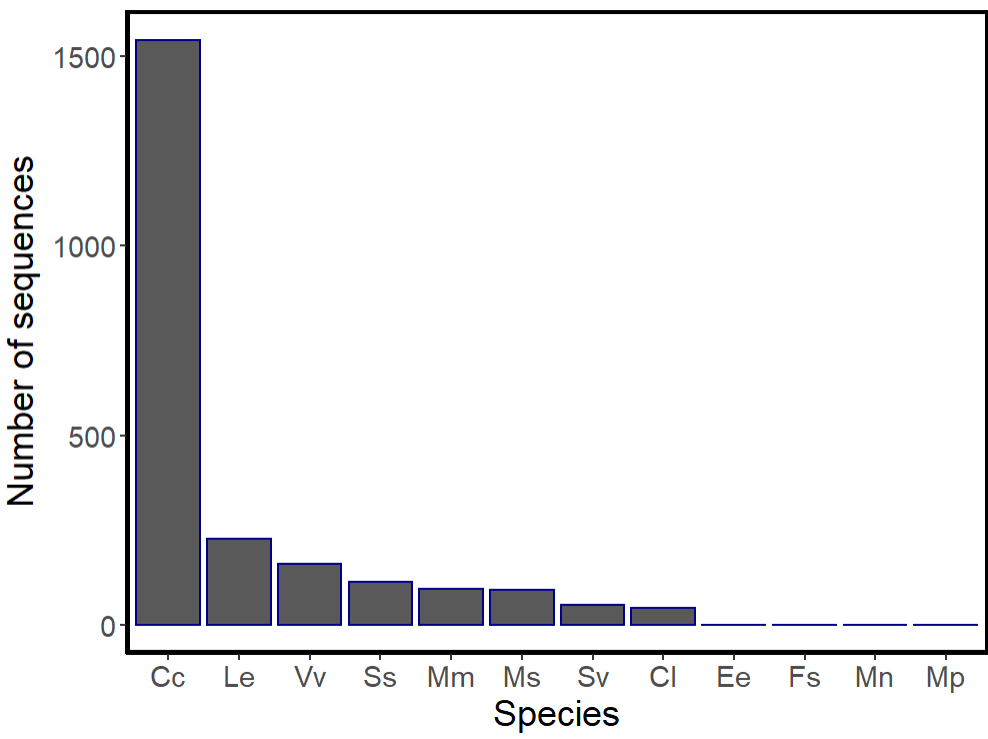
**

**Figure 1 S2**: Frequency plot of the number of medium-size mammals independent sequences recorded. Periods longer than 10 minutes were selected to consider two consecutive sequences of the same species in the same camera as an independent. Micro-mammals (mainly rodents) were not included in the plot due to the difficulties to recognize the species. A total of 402 bird sequences were also recorded. Abbreviations: Cc (*Capreolus capreolus*), Le (*Lepus europaeus*), Vv (*Vulpes vulpes*), Ss (*Sus scrofa*), Mm (*Meles meles*), Ms (*Martes martes and Martes foina*), Sv (*Sciurus vulgaris*), Cl (*Canis lupus*), Ee (*Erinaceus europaeus*), Fs (*Felis silvestris*), Mn (*Mustela nivalis*) and Mp (*Mustela putorious*).

Appendix S3: row data from camera traps

**Table 1 S3:** Raw data from roe deer (*Capreolus capreolus*), wild boar (*Sus scrofa*) and wolf (*Canis lupus*) to replicate random encounter model analysis.

| **Point_ID** | **Sp** | **G_size** | **Date** | **H_first** | **Speed (m/s)** | **Dist_det (m)** | **Ang_det (°)** |
| --- | --- | --- | --- | --- | --- | --- | --- |
| 1 | RoeDeer | 1 | 16/03/2022 | 12:44:42 |  | 18 | -5 |
| 1 | RoeDeer | 2 | 17/03/2022 | 18:33:56 |  | 4.7 | 23 |
| 1 | RoeDeer |  | 17/03/2022 |  |  | 6 | 23 |
| 1 | RoeDeer | 1 | 19/03/2022 | 11:32:32 |  | 12 | -25 |
| 1 | RoeDeer | 1 | 22/03/2022 | 12:26:45 |  | 13 | 21 |
| 2 | RoeDeer | 1 | 11/03/2022 | 3:06:02 |  | 13 | 20 |
| 2 | RoeDeer | 1 | 12/03/2022 | 13:07:57 | 0.24 | 4.8 | -26 |
| 2 | RoeDeer | 1 | 14/03/2022 | 13:08:35 | 3.50 | 6.5 | 8 |
| 2 | RoeDeer | 1 | 15/03/2022 | 16:58:07 | 0.27 | 12 | -6 |
| 2 | RoeDeer | 1 | 17/03/2022 | 14:36:19 | 0.14 | 6.5 | 27 |
| 2 | RoeDeer | 1 | 23/03/2022 | 11:34:25 | 0.07 | 5.3 | 26 |
| 2 | RoeDeer |  | 23/03/2022 | 11:36:06 | 0.12 | 5.2 | 27 |
| 2 | RoeDeer | 1 | 25/03/2022 | 9:42:56 | 0.16 | 8.5 | -3 |
| 2 | RoeDeer | 1 | 02/04/2022 | 3:50:43 |  | 12 | 4 |
| 2 | RoeDeer | 1 | 03/04/2022 | 4:26:50 | 0.02 | 6.1 | 18 |
| 2 | RoeDeer | 1 | 07/04/2022 | 6:52:50 | 0.22 | 6.6 | 27 |
| 2 | RoeDeer | 1 | 08/04/2022 | 4:06:14 | 0.48 | 7.8 | 14 |
| 2 | RoeDeer | 1 | 11/04/2022 | 4:17:03 | 1.07 | 8.5 | 7 |
| 2 | RoeDeer | 1 | 12/04/2022 | 6:55:15 | 0.19 | 9.5 | 8 |
| 2 | RoeDeer | 1 | 12/04/2022 | 9:13:05 | 1.28 | 8.3 | 18 |
| 2 | RoeDeer | 1 | 14/04/2022 | 6:40:53 |  | 8.5 | 22 |
| 2 | RoeDeer | 1 | 14/04/2022 | 9:46:49 | 0.10 | 7.3 | 4 |
| 2 | RoeDeer | 1 | 15/04/2022 | 7:07:46 | 0.10 | 5.4 | 21 |
| 2 | RoeDeer |  | 15/04/2022 | 7:09:30 |  | 8 | 25 |
| 2 | RoeDeer |  | 15/04/2022 | 7:11:38 | 0.36 | 9 | 22 |
| 2 | RoeDeer | 1 | 18/04/2022 | 7:12:14 | 0.05 | 6.9 | 14 |
| 2 | RoeDeer | 1 | 18/04/2022 | 9:53:26 | 0.25 | 8.6 | 27 |
| 2 | RoeDeer | 1 | 20/04/2022 | 0:21:48 |  | 1.2 | -27 |
| 2 | RoeDeer | 1 | 22/04/2022 | 15:49:36 | 0.25 | 8.3 | 27 |
| 2 | RoeDeer | 1 | 23/04/2022 | 11:10:28 |  | 8.1 | 17 |
| 2 | RoeDeer | 1 | 24/04/2022 | 5:52:50 | 1.20 | 9.5 | 15 |
| 2 | RoeDeer | 1 | 25/04/2022 | 4:30:32 |  | 5.2 | -16 |
| 2 | RoeDeer |  | 25/04/2022 | 4:31:36 |  | 6 | -26 |
| 2 | RoeDeer | 2 | 25/04/2022 | 18:31:52 | 0.13 | 8 | -18 |
| 2 | RoeDeer |  | 25/04/2022 | 18:31:52 |  | 12 | 20 |
| 2 | RoeDeer | 1 | 26/04/2022 | 5:19:29 | 0.10 | 5.6 | -21 |
| 2 | RoeDeer | 1 | 27/04/2022 | 5:27:09 | 1.20 | 9.5 | 17 |
| 2 | RoeDeer | 1 | 27/04/2022 | 6:03:28 | 0.12 | 4.3 | 23 |
| 3 | RoeDeer | 1 | 18/03/2022 | 6:54:00 | 0.03 | 5.2 | -25 |
| 3 | RoeDeer | 1 | 18/03/2022 | 18:11:53 |  | 8.8 | -4 |
| 3 | RoeDeer | 1 | 19/03/2022 | 7:04:07 |  | 3.4 | 22 |
| 3 | RoeDeer | 1 | 19/03/2022 | 12:04:27 |  | 40 | 21 |
| 3 | RoeDeer | 1 | 20/03/2022 | 10:45:10 | 0.50 | 3.1 | 22 |
| 3 | RoeDeer | 1 | 21/03/2022 | 7:34:15 |  | 9 | -23 |
| 3 | RoeDeer | 2 | 24/03/2022 | 7:35:17 | 3.00 | 8.5 | -18 |
| 3 | RoeDeer |  | 24/03/2022 | 7:35:17 | 3.17 | 10.5 | -25 |
| 3 | RoeDeer | 1 | 25/03/2022 | 11:51:10 | 0.17 | 9 | -26 |
| 3 | RoeDeer | 1 | 25/03/2022 | 14:35:53 |  | 9 | -27 |
| 3 | RoeDeer | 1 | 26/03/2022 | 6:24:50 | 0.09 | 4.3 | 27 |
| 3 | RoeDeer |  | 26/03/2022 | 6:26:35 | 0.10 | 8 | 27 |
| 3 | RoeDeer | 1 | 26/03/2022 | 8:30:14 | 2.15 | 8.2 | -18 |
| 3 | RoeDeer | 1 | 26/03/2022 | 16:30:43 |  | 7.6 | 4 |
| 3 | RoeDeer | 1 | 29/03/2022 | 9:19:02 | 0.16 | 7.4 | 11 |
| 3 | RoeDeer | 1 | 02/04/2022 | 7:20:42 | 0.12 | 1.5 | -27 |
| 3 | RoeDeer | 1 | 05/04/2022 | 8:46:22 | 0.06 | 7 | 26 |
| 3 | RoeDeer | 1 | 15/04/2022 | 18:50:22 | 0.08 | 6.5 | -12 |
| 3 | RoeDeer | 1 | 16/04/2022 | 20:14:39 |  | 6.7 | 16 |
| 4 | RoeDeer | 1 | 21/04/2022 | 5:53:19 | 0.06 | 7.7 | -12 |
| 4 | RoeDeer | 1 | 21/04/2022 | 16:31:03 |  | 5.3 | -27 |
| 4 | RoeDeer | 1 | 24/04/2022 | 7:46:42 |  | 12 | -12 |
| 6 | RoeDeer | 1 | 15/04/2022 | 8:01:00 |  | 7.6 | -16 |
| 6 | RoeDeer | 1 | 16/04/2022 | 11:29:51 | 0.64 | 7.2 | -25 |
| 7 | RoeDeer | 1 | 19/03/2022 | 4:19:03 |  | 2 | 21 |
| 7 | RoeDeer | 1 | 23/03/2022 | 18:41:12 |  | 15 | -2 |
| 7 | RoeDeer | 1 | 27/03/2022 | 10:08:41 |  | 1 | -25 |
| 7 | RoeDeer | 1 | 30/03/2022 | 6:28:29 |  | 18 | 6 |
| 7 | RoeDeer | 1 | 11/04/2022 | 23:14:51 | 0.19 | 8.2 | 4 |
| 7 | RoeDeer | 2 | 13/04/2022 | 16:26:50 |  | 15 | 12 |
| 7 | RoeDeer | 1 | 21/04/2022 | 16:23:11 |  | 3 | 27 |
| 8 | RoeDeer | 1 | 24/03/2022 | 6:23:27 |  | 2.3 | 20 |
| 8 | RoeDeer | 1 | 17/04/2022 | 8:25:41 |  | 8 | -18 |
| 11 | RoeDeer | 1 | 18/03/2022 | 6:47:08 | 0.07 | 4.2 | 26 |
| 11 | RoeDeer | 1 | 20/03/2022 | 22:56:00 |  | 6 | -1 |
| 11 | RoeDeer | 1 | 26/03/2022 | 11:39:58 | 0.04 | 4.5 | 6 |
| 11 | RoeDeer | 1 | 26/03/2022 | 12:30:19 | 0.25 | 2 | -27 |
| 11 | RoeDeer | 1 | 29/03/2022 | 6:51:59 | 0.23 | 4.6 | -25 |
| 11 | RoeDeer | 1 | 01/04/2022 | 17:30:47 | 0.33 | 3.2 | -27 |
| 11 | RoeDeer | 1 | 02/04/2022 | 21:55:27 | 0.22 | 5 | -25 |
| 11 | RoeDeer | 1 | 04/04/2022 | 21:05:08 | 0.14 | 1.7 | -16 |
| 11 | RoeDeer | 1 | 06/04/2022 | 0:28:30 | 0.25 | 3.6 | -27.5 |
| 11 | RoeDeer | 1 | 08/04/2022 | 19:53:47 | 1.20 | 1.7 | 12 |
| 11 | RoeDeer | 1 | 16/04/2022 | 6:51:42 | 0.05 | 5.2 | -2 |
| 11 | RoeDeer |  | 16/04/2022 | 6:54:24 | 0.18 | 3.7 | -23 |
| 11 | RoeDeer | 1 | 19/04/2022 | 6:12:38 | 0.11 | 3.1 | 1 |
| 11 | RoeDeer |  | 19/04/2022 | 6:13:26 |  | 2.7 | 13 |
| 11 | RoeDeer | 2 | 20/04/2022 | 1:22:38 | 1.80 | 5.9 | -17 |
| 11 | RoeDeer |  | 20/04/2022 | 1:22:52 |  | 3.7 | -16 |
| 13 | RoeDeer | 1 | 19/04/2022 | 23:43:44 | 3.15 | 8 | -11 |
| 13 | RoeDeer | 1 | 20/04/2022 | 3:24:14 | 1.20 | 3.1 | -27 |
| 14 | RoeDeer | 1 | 06/04/2022 | 6:27:15 |  | 7.7 | -7 |
| 14 | RoeDeer | 1 | 14/04/2022 | 18:55:31 | 1.00 | 7.7 | 25 |
| 14 | RoeDeer | 1 | 18/04/2022 | 20:06:08 | 2.20 | 9 | 17 |
| 14 | RoeDeer | 1 | 29/03/2022 | 10:44:38 |  | 6.5 | -23 |
| 14 | RoeDeer |  | 29/03/2022 | 10:53:45 |  | 1.8 | 18 |
| 14 | RoeDeer | 1 | 19/04/2022 | 10:33:59 |  | 2.6 | -27 |
| 14 | RoeDeer |  | 19/04/2022 | 10:35:20 |  | 1.7 | 27 |
| 16 | RoeDeer | 1 | 13/03/2022 | 13:10:21 |  | 15 | 12 |
| 16 | RoeDeer | 2 | 17/03/2022 | 6:54:50 |  | 7 | -2 |
| 16 | RoeDeer |  | 17/03/2022 | 6:54:50 |  | 12 | 0 |
| 16 | RoeDeer | 1 | 18/03/2022 | 16:46:10 |  | 15 | -9 |
| 16 | RoeDeer | 1 | 18/03/2022 | 18:31:49 | 0.25 | 5.8 | 23 |
| 16 | RoeDeer | 1 | 20/03/2022 | 13:16:13 | 0.20 | 2.7 | 27 |
| 16 | RoeDeer | 1 | 22/03/2022 | 7:32:37 |  | 15 | 15 |
| 16 | RoeDeer | 1 | 25/03/2022 | 9:46:34 |  | 8.5 | 17 |
| 16 | RoeDeer | 1 | 26/03/2022 | 6:32:38 | 0.11 | -13 | 13 |
| 16 | RoeDeer | 1 | 28/03/2022 | 8:59:43 | 0.26 | -9 | 9.2 |
| 16 | RoeDeer | 1 | 04/04/2022 | 17:09:54 | 0.26 | 11 | 14 |
| 16 | RoeDeer | 1 | 09/04/2022 | 8:49:13 |  | 8.3 | -21 |
| 16 | RoeDeer | 1 | 09/04/2022 | 13:38:09 |  | 11.5 | -3 |
| 16 | RoeDeer | 1 | 12/04/2022 | 11:56:00 | 0.05 | 2.1 | 23 |
| 16 | RoeDeer | 1 | 13/04/2022 | 19:58:43 | 0.05 | 3.2 | 27 |
| 16 | RoeDeer | 1 | 14/04/2022 | 19:07:09 | 0.14 | 8.5 | 17 |
| 16 | RoeDeer | 1 | 14/04/2022 | 21:40:55 | 0.01 | 3.5 | 27.5 |
| 16 | RoeDeer | 1 | 16/04/2022 | 18:31:35 | 0.06 | 7 | 24 |
| 16 | RoeDeer | 1 | 24/04/2022 | 18:36:32 | 0.04 | 5 | -26 |
| 16 | RoeDeer | 1 | 24/04/2022 | 12:56:31 | 0.16 | 9.4 | -9 |
| 16 | RoeDeer | 1 | 24/04/2022 | 21:17:38 | 0.09 | 1.8 | 27 |
| 16 | RoeDeer | 1 | 26/04/2022 | 17:23:49 | 0.16 | 11.5 | 7 |
| 18 | RoeDeer | 1 | 22/03/2022 | 17:07:49 |  | 5.2 | -24 |
| 18 | RoeDeer | 1 | 24/03/2022 | 19:27:39 |  | 6.6 | 18 |
| 20 | RoeDeer | 1 | 25/03/2022 | 9:23:46 |  | 3.3 | 19 |
| 20 | RoeDeer | 1 | 27/03/2022 | 6:53:30 | 0.07 | 1.8 | 27.5 |
| 20 | RoeDeer | 1 | 06/04/2022 | 12:52:58 | 0.17 | 0.6 | 27.5 |
| 23 | RoeDeer | 1 | 20/03/2022 | 4:44:12 | 0.15 | 5.7 | -22 |
| 23 | RoeDeer | 1 | 22/03/2022 | 22:44:18 | 0.07 | 6.8 | 16 |
| 23 | RoeDeer | 1 | 22/03/2022 | 23:56:19 |  | 3.8 | 16 |
| 23 | RoeDeer | 1 | 24/03/2022 | 19:31:55 | 0.01 | 5.2 | -27.5 |
| 24 | RoeDeer | 2 | 18/03/2022 | 6:51:33 |  | 2.8 | 27 |
| 24 | RoeDeer |  | 18/03/2022 | 6:52:05 | 0.16 | 2.6 | -3 |
| 24 | RoeDeer | 1 | 18/03/2022 | 9:49:37 | 0.01 | 2.1 | -27.5 |
| 24 | RoeDeer | 1 | 20/03/2022 | 12:04:36 |  | 2.6 | -12 |
| 24 | RoeDeer | 1 | 22/03/2022 | 15:10:46 | 0.05 | 2.9 | 27.5 |
| 24 | RoeDeer | 1 | 23/03/2022 | 23:04:14 |  | 6 | 23 |
| 25 | RoeDeer | 1 | 02/04/2022 | 21:56:33 | 0.40 | 1.2 | -27.5 |
| 25 | RoeDeer | 1 | 08/04/2022 | 7:32:09 | 0.54 | 2.1 | -19 |
| 26 | RoeDeer | 1 | 06/04/2022 | 2:15:06 |  | 0.5 | 27.5 |
| 26 | RoeDeer | 1 | 08/04/2022 | 14:37:59 |  | 15 | -4 |
| 26 | RoeDeer | 1 | 09/04/2022 | 2:18:47 |  | 16 | -15 |
| 26 | RoeDeer | 1 | 09/04/2022 | 13:00:15 |  | 12 | 21 |
| 26 | RoeDeer | 1 | 12/04/2022 | 6:51:35 |  | 20 | -6 |
| 26 | RoeDeer | 1 | 13/04/2022 | 8:51:50 |  | 20 | 6 |
| 26 | RoeDeer | 1 | 20/04/2022 | 19:34:45 |  | 8 | -7 |
| 27 | RoeDeer | 1 | 18/03/2022 | 10:44:07 |  | 5.3 | 15 |
| 27 | RoeDeer | 1 | 19/03/2022 | 22:03:04 |  | 3.2 | 15 |
| 27 | RoeDeer | 1 | 20/03/2022 | 1:51:50 | 0.44 | 6 | 25 |
| 27 | RoeDeer | 1 | 21/03/2022 | 4:33:59 | 0.15 | 6 | 24 |
| 27 | RoeDeer | 1 | 21/03/2022 | 6:37:16 | 0.08 | 3.1 | 23 |
| 27 | RoeDeer | 1 | 29/03/2022 | 20:12:50 |  | 5.3 | -8 |
| 27 | RoeDeer | 1 | 30/03/2022 | 5:02:19 | 0.32 | 6.5 | 24 |
| 27 | RoeDeer | 1 | 31/03/2022 | 7:28:35 | 2.15 | 4.8 | -27.5 |
| 27 | RoeDeer | 1 | 01/04/2022 | 6:33:22 | 0.40 | 7.6 | 13 |
| 27 | RoeDeer | 1 | 03/04/2022 | 18:31:20 | 0.36 | 4.9 | 14 |
| 27 | RoeDeer | 1 | 07/04/2022 | 13:20:35 | 0.13 | 5.4 | 23 |
| 27 | RoeDeer | 1 | 08/04/2022 | 3:10:53 | 0.51 | 2 | 27.5 |
| 27 | RoeDeer | 1 | 09/04/2022 | 16:01:09 | 0.37 | 8.5 | 12 |
| 27 | RoeDeer | 1 | 11/04/2022 | 19:33:16 |  | 11 | -5 |
| 27 | RoeDeer | 1 | 13/04/2022 | 11:45:48 |  | 5.4 | 15 |
| 27 | RoeDeer | 1 | 14/04/2022 | 18:32:13 | 0.21 | 5.5 | 27.5 |
| 27 | RoeDeer | 1 | 19/04/2022 | 18:21:21 | 0.35 | 14 | 14 |
| 27 | RoeDeer | 1 | 19/04/2022 | 18:48:20 | 0.24 | 4.8 | -27.5 |
| 27 | RoeDeer | 1 | 20/04/2022 | 18:46:09 | 0.20 | 2.3 | -19 |
| 27 | RoeDeer |  | 20/04/2022 | 18:47:12 |  | 9.5 | 21 |
| 27 | RoeDeer | 1 | 21/04/2022 | 17:56:28 | 0.37 | 8.4 | 17 |
| 27 | RoeDeer | 1 | 21/04/2022 | 10:26:56 |  | 12 | -21 |
| 27 | RoeDeer | 1 | 21/04/2022 | 10:45:18 | 0.75 | 2 | 0 |
| 27 | RoeDeer | 1 | 22/04/2022 | 5:32:10 | 0.90 | 3.2 | -27.5 |
| 27 | RoeDeer | 1 | 22/04/2022 | 11:51:35 |  | 5.8 | 11 |
| 28 | RoeDeer | 1 | 13/03/2022 | 18:13:55 |  | 1.3 | -27.5 |
| 28 | RoeDeer | 1 | 18/03/2022 | 18:11:17 |  | 5.2 | -13 |
| 28 | RoeDeer | 1 | 22/03/2022 | 13:26:53 |  | 15 | 27 |
| 28 | RoeDeer | 1 | 23/03/2022 | 20:21:44 |  | 1 | -27.5 |
| 30 | RoeDeer | 1 | 24/03/2022 | 19:09:22 |  | 7 | 3 |
| 30 | RoeDeer | 1 | 28/03/2022 | 9:37:48 |  | 3.1 | -24 |
| 30 | RoeDeer | 1 | 29/03/2022 | 22:05:51 |  | 8 | 4 |
| 30 | RoeDeer | 1 | 10/04/2022 | 6:12:47 | 1.02 | 3.3 | -14 |
| 30 | RoeDeer | 1 | 12/04/2022 | 11:17:10 |  | 1.8 | 27.5 |
| 30 | RoeDeer | 1 | 12/04/2022 | 17:39:37 |  | 8.5 | 6 |
| 30 | RoeDeer | 1 | 18/04/2022 | 6:18:22 | 0.58 | 4 | -1 |
| 32 | RoeDeer | 1 | 12/03/2022 | 8:53:19 |  | 7 | -13 |
| 32 | RoeDeer | 1 | 18/03/2022 | 12:50:09 | 0.30 | 3.7 | -25 |
| 32 | RoeDeer | 1 | 18/03/2022 | 17:27:46 |  | 7 | -15 |
| 32 | RoeDeer | 1 | 22/03/2022 | 18:26:08 | 0.12 | 4.5 | -14 |
| 33 | RoeDeer | 1 | 19/03/2022 | 13:20:07 | 0.26 | 7.6 | -12 |
| 33 | RoeDeer | 1 | 27/04/2022 | 1:32:26 | 0.04 | 5.4 | -17 |
| 35 | RoeDeer | 1 | 11/03/2022 | 22:39:08 |  | 7.8 | -14 |
| 35 | RoeDeer | 1 | 14/03/2022 | 21:30:16 |  | 3.2 | 12 |
| 39 | RoeDeer | 1 | 20/03/2022 | 7:23:54 |  | 5.2 | -26 |
| 39 | RoeDeer | 1 | 21/03/2022 | 18:23:04 |  | 11 | 23 |
| 39 | RoeDeer |  | 21/03/2022 | 18:23:42 |  | 15 | 27 |
| 39 | RoeDeer | 1 | 15/04/2022 | 14:43:31 | 0.30 | 2.6 | -19 |
| 39 | RoeDeer | 1 | 20/04/2022 | 13:06:22 |  | 7 | 24 |
| 39 | RoeDeer | 1 | 22/04/2022 | 7:42:01 |  | 3.8 | 24 |
| 40 | RoeDeer | 1 | 18/03/2022 | 20:09:44 |  | 2.7 | 15 |
| 2 | WildBoar | 1 | 15/03/2022 | 5:53:54 | 0.12 | 5.8 | 24 |
| 2 | WildBoar | 1 | 07/04/2022 | 8:10:51 | 0.43 | 5.3 | 22 |
| 2 | WildBoar | 1 | 07/04/2022 | 5:22:39 | 0.8 | 2.2 | -13 |
| 27 | WildBoar | 1 | 23/03/2022 | 18:22:14 | 2.2 | 6.4 | -22 |
| 27 | WildBoar | 1 | 27/03/2022 | 5:44:25 |  | 5.2 | -3 |
| 27 | WildBoar | 1 | 28/03/2022 | 17:05:26 | 2.27 | 6.8 | -23 |
| 27 | WildBoar | 1 | 30/03/2022 | 17:49:31 | 1.4 | 7.8 | -26 |
| 27 | WildBoar | 1 | 02/04/2022 | 8:15:51 |  | 0.5 | 10 |
| 27 | WildBoar | 1 | 22/04/2022 | 6:25:02 | 0.35 | 1.7 | -22 |
| 27 | WildBoar | 1 | 27/04/2022 | 4:57:44 | 0.85 | 1.1 | -27 |
| 27 | WildBoar | 1 | 24/03/2022 | 23:04:29 |  | 6.5 | 0 |
| 5 | WildBoar | 1 | 11/03/2022 | 21:14:35 | 0.58 | 1 | -27 |
| 30 | WildBoar | 1 | 27/03/2022 | 4:48:39 |  | 6.8 | 8 |
| 25 | WildBoar | 1 | 21/03/2022 | 12:40:56 |  | 0.5 | 0 |
| 25 | WildBoar | 1 | 15/04/2022 | 9:57:05 | 0.35 | 2.4 | 22 |
| 9 | WildBoar | 1 | 20/03/2020 | 18:47:26 | 0.13 | 6.3 | 17 |
| 9 | WildBoar |  | 20/03/2020 | 18:49:15 |  | 8 | 22 |
| 39 | WildBoar | 1 | 15/04/2022 | 1:03:12 | 0.28 | 2.2 | 27 |
| 33 | WildBoar | 1 | 12/04/2022 | 1:29:26 | 0.7 | 7.6 | -16 |
| 26 | WildBoar | 1 | 21/03/2022 | 10:31:56 | 0.27 | 9.7 | -2 |
| 37 | WildBoar | 1 | 18/03/2022 | 7:13:31 | 0.43 | 7.8 | -4 |
| 5 | WildBoar | 2 | 04/05/2022 | 5:02:13 |  | 25 | 10 |
| 5 | WildBoar |  | 04/05/2022 | 5:02:31 | 0.11 | 5.3 | -27 |
| 5 | WildBoar | 1 | 19/05/2022 | 21:53:07 | 0.85 | 1.7 | 0 |
| 11 | WildBoar | 1 | 27/04/2022 | 23:19:41 | 0.85 | 5.3 | 6 |
| 11 | WildBoar | 1 | 28/04/2022 | 20:51:26 | 0.6 | 5.4 | -24 |
| 11 | WildBoar | 1 | 06/05/2022 | 16:25:34 | 0.1 | 7 | -2 |
| 11 | WildBoar | 1 | 19/05/2022 | 20:19:46 | 0.08 | 4.7 | 21 |
| 11 | WildBoar |  | 19/05/2022 | 20:24:32 | 0.05 | 3.8 | -22 |
| 11 | WildBoar |  | 19/05/2022 | 20:28:00 | 0.25 | 4 | 26 |
| 11 | WildBoar | 1 | 21/05/2022 | 18:58:30 | 1 | 7.6 | 8 |
| 11 | WildBoar | 1 | 26/05/2022 | 12:39:08 | 0.1 | 5.2 | -23 |
| 20 | WildBoar | 1 | 30/05/2022 | 10:15:26 | 3.25 | 12 | 18 |
| 27 | WildBoar | 1 | 28/04/2022 | 7:09:59 |  | 2.3 | 13 |
| 27 | WildBoar | 1 | 02/05/2022 | 10:50:42 |  | 1 | 0 |
| 27 | WildBoar | 1 | 04/05/2022 | 6:42:19 |  | 4.7 | 25 |
| 27 | WildBoar |  | 04/05/2022 | 6:43:06 |  | 3.2 | 9 |
| 27 | WildBoar | 1 | 12/05/2022 | 7:18:46 | 1.25 | 5.8 | -17 |
| 27 | WildBoar | 1 | 29/05/2022 | 18:19:33 | 1.2 | 5.2 | 18 |
| 35 | WildBoar | 1 | 26/05/2022 | 5:56:32 |  | 1 | -10 |
| 1 | WildBoar | 1 | 27/04/2022 | 20:16:03 |  | 0.5 | 0 |
| 1 | WildBoar |  | 27/04/2022 |  |  | 3 | 27 |
| 1 | WildBoar |  | 27/04/2022 | 20:16:44 | 0.45 | 6 | -18 |
| 1 | WildBoar | 1 | 06/05/2022 | 14:52:30 | 0.01 | 9.5 | -17 |
| 1 | WildBoar | 2 | 06/05/2022 | 15:23:21 | 0.03 | 2.3 | -21 |
| 1 | WildBoar |  | 06/05/2022 | 15:24:21 | 0.24 | 1.5 | -18 |
| 1 | WildBoar | 2 | 06/05/2022 | 15:36:45 |  | 12 | -17 |
| 14 | WildBoar | 1 | 14/05/2022 | 6:22:48 | 3 | 9.8 | 12 |
| 14 | WildBoar | 1 | 28/05/2022 | 6:06:12 | 3.8 | 4.7 | -9 |
| 14 | WildBoar | 1 | 23/04/2022 | 5:25:02 |  | 16 | 18 |
| 14 | WildBoar | 1 | 24/05/2022 | 20:42:25 |  | 22 | -2 |
| 24 | WildBoar | 1 | 05/05/2022 | 18:52:26 | 1.3 | 8.5 | 24 |
| 11 | WildBoar | 1 | 22/06/2022 | 3:07:03 | 0.9 | 5.81 | -19 |
| 11 | WildBoar | 1 | 01/07/2022 | 10:46:51 | 1.07 | 9.77 | 6 |
| 18 | WildBoar | 1 | 30/06/2022 | 17:33:19 | 0.21 | 7.74 | -7 |
| 2 | WildBoar | 1 | 17/06/2022 | 21:19:38 |  | 4.07 | 14 |
| 20 | WildBoar | 1 | 10/06/2022 | 3:58:00 | 1.5 | 2.8 | 0 |
| 24 | WildBoar | 1 | 01/07/2022 | 0:33:53 | 0.58 | 6.05 | 23 |
| 27 | WildBoar | 2 | 07/06/2022 | 7:40:22 | 1.61 | 6.88 | -5 |
| 27 | WildBoar |  | 07/06/2022 | 7:41:13 | 0.94 | 5.59 | 11 |
| 27 | WildBoar | 1 | 27/06/2022 | 15:20:50 | 0.8 | 8.56 | -18 |
| 29 | WildBoar | 1 | 12/06/2022 | 4:24:01 |  | 4.7 | 22 |
| 3 | WildBoar | 1 | 10/06/2022 | 0:54:02 | 0.84 | 12.55 | 4 |
| 3 | WildBoar | 1 | 14/06/2022 | 21:50:05 | 0.25 | 12.45 | 19 |
| 32 | WildBoar | 1 | 29/06/2022 | 21:11:22 | 2.31 | 14.76 | -3 |
| 35 | WildBoar | 1 | 01/06/2022 | 20:45:36 | 1.17 | 9.36 | 1 |
| 35 | WildBoar | 1 | 27/06/2022 | 20:03:59 | 0.69 | 8.61 | 19 |
| 5 | WildBoar | 1 | 19/06/2022 | 2:41:27 | 0.22 | 3.23 | 18 |
| 5 | WildBoar | 1 | 23/06/2022 | 21:07:04 | 0.26 | 7.65 | 18 |
| 5 | WildBoar |  | 23/06/2022 | 21:07:29 | 1 | 3.81 | 21 |
| 1 | WildBoar | 2 | 28/07/2022 | 20:10:12 | 0.21 | 12 | -15.21 |
| 1 | WildBoar |  | 28/07/2022 | 20:10:16 |  | 12.5 | -17.9 |
| 1 | WildBoar | 2 | 28/07/2022 | 17:41:08 |  | 12 | -20 |
| 1 | WildBoar |  | 28/07/2022 | 17:41:08 |  | 12.5 | -19 |
| 18 | WildBoar | 1 | 24/07/2022 | 20:26:32 | 0.53 | 2.8 | 20 |
| 20 | WildBoar | 1 | 11/07/2022 | 20:46:19 | 0.49 | 2.7 | -0.24 |
| 24 | WildBoar | 1 | 08/07/2022 | 1:53:57 | 1.03 | 4.8 | 22.88 |
| 24 | WildBoar | 1 | 21/07/2022 | 20:59:27 | 0.81 | 6.2 | -19.53 |
| 27 | WildBoar | 1 | 09/07/2022 | 6:43:19 | 0.37 | 10.16 | -2.91 |
| 27 | WildBoar | 1 | 31/07/2022 | 12:17:26 | 1.1 | 9.79 | -8.93 |
| 3 | WildBoar | 1 | 11/07/2022 | 3:07:30 | 0.85 | 4.7 | 21.01 |
| 3 | WildBoar | 1 | 23/07/2022 | 22:47:05 | 1.28 | 12.86 | 17.45 |
| 32 | WildBoar | 1 | 28/07/2022 | 4:54:22 | 0.64 | 1.13 | 28.25 |
| 35 | WildBoar | 1 | 12/07/2022 | 6:05:50 | 1.04 | 15.84 | 13.06 |
| 35 | WildBoar | 1 | 12/07/2022 | 6:27:58 | 2.83 | 6.99 | 18.8 |
| 35 | WildBoar | 1 | 19/07/2022 | 3:49:57 | 3.16 | 16.78 | 12.43 |
| 5 | WildBoar | 2 | 08/07/2022 | 20:29:11 | 0.18 | 7.91 | 3.98 |
| 5 | WildBoar |  | 08/07/2022 | 20:30:42 |  | 1.34 | 14.84 |
| 5 | WildBoar |  | 08/07/2022 | 20:32:19 | 0.1 | 4.24 | -22.01 |
| 5 | WildBoar |  | 08/07/2022 | 20:33:34 | 0.31 | 11.32 | 18.73 |
| 5 | WildBoar | 1 | 21/07/2022 | 6:06:26 | 0.62 | 6.83 | 6.33 |
| 5 | WildBoar |  | 21/07/2022 | 6:07:07 | 1.28 | 1.95 | -17.36 |
| 5 | WildBoar | 2 | 05/08/2022 | 4:35:19 | 0.69 | 8.95 | -12.35 |
| 2 | Wolf | 1 | 12/03/2022 | 5:08:14 | 0.33 | 5.8 | 26 |
| 4 | Wolf | 1 | 26/04/2022 | 19:02:09 | 0.31 | 12 | 6 |
| 9 | Wolf | 1 | 20/03/2022 | 22:32:20 |  | 6.9 | 22 |
| 24 | Wolf | 1 | 24/03/2022 | 7:44:52 |  | 2 | 0 |
| 24 | Wolf | 1 | 02/04/2022 | 5:37:00 | 2.90 | 3.2 | -15 |
| 27 | Wolf | 3 | 21/03/2022 | 9:28:25 | 0.78 | 4.6 | 20 |
| 27 | Wolf |  | 21/03/2022 | 9:28:36 |  |  |  |
| 27 | Wolf |  | 21/03/2022 | 9:28:52 | 2.50 | 6.2 | 20 |
| 39 | Wolf | 1 | 24/03/2022 | 19:52:34 |  | 2.6 | -21 |
| 39 | Wolf | 1 | 27/03/2022 | 22:39:08 | 2.10 | 2.5 | -21 |
| 39 | Wolf | 1 | 28/03/2022 | 1:16:06 | 0.50 | 2.6 | -27 |
| 39 | Wolf | 1 | 30/03/2022 | 21:10:06 | 1.67 | 2.7 | -27 |
| 39 | Wolf | 1 | 22/04/2022 | 3:38:50 | 0.93 | 2.8 | -18 |
| 4 | Wolf | 1 | 05/05/2022 | 4:19:46 |  | 2.6 | 28 |
| 10 | Wolf | 1 | 29/04/2022 | 7:00:00 | 0.33 | 5.8 | 8 |
| 26 | Wolf | 2 | 09/05/2022 | 0:04:56 | 0.40 | 3.8 | -26 |
| 26 | Wolf |  | 09/05/2022 | 0:05:15 | 1.00 | 4 | -22 |
| 26 | Wolf |  | 09/05/2022 | 0:07:04 | 0.43 | 5.3 | 22 |
| 26 | Wolf |  | 09/05/2022 | 0:07:11 | 0.59 | 5.6 | 27 |
| 26 | Wolf | 2 | 09/05/2022 | 6:41:23 | 1.50 | 14 | -12 |
| 26 | Wolf |  | 09/05/2022 | 6:42:09 | 0.93 | 16 | -15 |
| 26 | Wolf | 1 | 21/05/2022 | 19:58:39 | 0.78 | 15 | -6 |
| 14 | Wolf | 1 | 28/04/2022 | 19:30:10 |  | 3.8 | -20 |
| 24 | Wolf | 1 | 14/05/2022 | 18:18:23 | 2.10 | 4.3 | 21 |
| 10 | Wolf | 2 | 22/06/2022 | 1:28:28 |  | 1.47 | -1 |
| 10 | Wolf |  | 22/06/2022 | 1:28:45 | 0.93 | 7.5 | 4 |
| 10 | Wolf | 1 | 23/06/2022 | 5:25:41 |  | 1.67 | 10 |
| 10 | Wolf | 1 | 02/06/2022 | 0:20:59 | 0.29 | 2.37 | 14 |
| 10 | Wolf | 2 | 04/06/2022 | 5:02:14 | 0.85 | 1 | 15 |
| 10 | Wolf |  | 04/06/2022 | 5:02:14 |  | 6.5 | 20 |
| 10 | Wolf |  | 04/06/2022 | 5:02:25 |  | 10.3 | -18 |
| 10 | Wolf | 1 | 04/06/2022 | 6:03:43 |  | 7.63 | 9 |
| 10 | Wolf | 1 | 04/06/2022 | 8:51:01 | 2.81 | 6.83 | 1 |
| 10 | Wolf | 1 | 04/06/2022 | 23:11:09 | 1.28 | 4.2 | -9 |
| 11 | Wolf | 1 | 14/06/2022 | 12:41:03 | 0.89 | 5.37 | -18 |
| 24 | Wolf | 1 | 07/06/2022 | 22:46:02 |  | 4.78 | -7 |
| 26 | Wolf | 1 | 28/06/2022 | 3:38:41 | 0.89 | 2.83 | -24 |
| 10 | Wolf | 1 | 09/07/2022 | 2:58:46 | 1.20 | 1.55 | -25.8 |
| 27 | Wolf | 1 | 31/07/2022 | 16:35:16 | 0.67 | 6.32 | 12.04 |
| 32 | Wolf | 1 | 13/07/2022 | 12:01:36 |  | 1.39 | -25.8 |
| 37 | Wolf | 2 | 01/08/2022 | 2:00:37 | 2.63 | 1.15 | -1.15 |
| 37 | Wolf |  | 01/08/2022 | 2:00:46 |  | 1 | -15 |

**Table 2 S3**: Operativity matrix for wild boar and wolf.

| CAM | Operative days |
| --- | --- |
| 1 | 73 |
| 2 | 149 |
| 3 | 131 |
| 4 | 71 |
| 5 | 101 |
| 6 | 117 |
| 7 | 136 |
| 8 | 129 |
| 9 | 70 |
| 10 | 58 |
| 11 | 136 |
| 12 | 70 |
| 13 | 142 |
| 14 | 72 |
| 15 | 131 |
| 16 | 82 |
| 17 | 97 |
| 18 | 131 |
| 19 | 13 |
| 20 | 134 |
| 21 | 123 |
| 22 | 104 |
| 23 | 45 |
| 24 | 117 |
| 25 | 144 |
| 26 | 136 |
| 27 | 144 |
| 28 | 63 |
| 29 | 82 |
| 30 | 130 |
| 31 | 2 |
| 32 | 115 |
| 33 | 129 |
| 34 | 101 |
| 35 | 106 |
| 36 | 90 |
| 37 | 79 |
| 38 | 79 |
| 39 | 38 |
| 40 | 67 |
| 41 | 100 |

**Table 3 S3**: Operativity matrix for roe deer.

| CAM | Operative days |
| --- | --- |
| 1 | 8 |
| 2 | 49 |
| 3 | 36 |
| 4 | 23 |
| 5 | 2 |
| 6 | 18 |
| 7 | 38 |
| 8 | 38 |
| 9 | 5 |
| 10 | 6 |
| 11 | 37 |
| 12 | 0 |
| 13 | 44 |
| 14 | 38 |
| 15 | 36 |
| 16 | 48 |
| 17 | 2 |
| 18 | 36 |
| 19 | 2 |
| 20 | 36 |
| 21 | 23 |
| 22 | 23 |
| 23 | 11 |
| 24 | 19 |
| 25 | 45 |
| 26 | 38 |
| 27 | 45 |
| 28 | 19 |
| 29 | 2 |
| 30 | 32 |
| 31 | 2 |
| 32 | 16 |
| 33 | 41 |
| 34 | 6 |
| 35 | 7 |
| 36 | 2 |
| 37 | 5 |
| 38 | 26 |
| 39 | 38 |
| 40 | 9 |
| 41 | 8 |

**Table 4 S3**: Recruitment data.

| **SEX** | **PIGLETS** |
| --- | --- |
| Unknown | 0 |
| Female | 3 |
| Female | 3 |
| Alone | 1 |
| Female | 3 |
| Female | 1 |
| Male | 0 |
| Male | 0 |
| Unknown | 0 |
| Female | 0 |
| Unknown | 0 |
| Unknown | 0 |
| Male | 0 |
| Unknown | 0 |
| Unknown | 0 |
| Female | 0 |
| Female | 0 |
| Alone | 1 |
| Alone | 2 |
| Female | 4 |
| Male | 0 |
| Male | 0 |
| Alone | 2 |
| Male | 0 |
| Male | 0 |
| Male | 0 |
| Unknown | 0 |
| Unknown | 0 |
| Female | 2 |
| Male | 0 |
| Unknown | 0 |
| Female | 0 |
| Alone | 1 |
| Alone | 1 |
| Alone | 1 |
| Alone | 2 |
| Female | 0 |
| Alone | 2 |
| Alone | 1 |
| Alone | 2 |
| Alone | 1 |
| Unknown | 0 |
| Unknown | 0 |
| Female | 4 |
| Male | 0 |
| Male | 0 |
| Male | 0 |
| Female | 0 |
| Male | 0 |
| Female | 0 |
| Unknown | 0 |
| Male | 0 |
| Male | 0 |
| Male | 0 |
| Unknown | 0 |
| Unknown | 0 |
| Alone | 1 |
| Alone | 1 |
| Alone | 1 |
| Alone | 2 |
| Female | 1 |
| Alone | 2 |
| Alone | 1 |
| Alone | 1 |
| Alone | 2 |
| Alone | 1 |
| Female | 0 |
| Female | 0 |
| Male | 0 |
| Alone | 2 |
| Alone | 1 |
| Alone | 2 |

**Table 5 S3:** Biosafety human restriction data.

| CAM | Specie | Dog | N_people |
| --- | --- | --- | --- |
| 4 | Human | no | 1 |
| 16 | Human | yes | 1 |
| 14 | Human | yes | 1 |
| 14 | Human | yes | 1 |
| 12a | Human |  | stolen |
| 14 | Human |  | stolen |
| 16 | Human |  | stolen |
| 19 | Human |  | stolen |
| 23 | Human |  | stolen |
| 39a | Human |  | stolen |
| 39b | Human |  | stolen |
| 4 | Human | no | 1 |
| 5 | Human | yes | 1 |
| 5 | Human | yes | 0 |
| 6 | Human | no | 1 |
| 10 | Human | no | 2 |
| 10 | Human | yes | 1 |
| 13 | Human | yes | 2 |
| 13 | Human | no | 2 |
| 16 | Human | yes | 1 |
| 16 | Human | yes | 1 |
| 18 | Human | no | 2 |
| 29 | Human | no | 1 |
| 38 | Human | no | 1 |
| 3 | Human | no | 1 |
| 6 | Human | no | 1 |
| 13 | Human | no | 3 |
| 13 | Human | no | 4 |
| 13 | Human | no | 1 |
| 32 | Human | no | 1 |
| 7 | Human | yes | 0 |
| 3 | Human | yes | 0 |
| 30 | Human | yes | 0 |
| 5 | Human | yes | 1 |
| 18 | Human | no | 1 |
| 3 | Human | no | 1 |
| 13 | Human | no | 2 |
